# Tumour cell contamination of autologous stem cells grafts in high-risk neuroblastoma: the good news?

**DOI:** 10.1038/sj.bjc.6601014

**Published:** 2003-06-10

**Authors:** R Handgretinger, W Leung, K Ihm, P Lang, T Klingebiel, D Niethammer

**Affiliations:** 1 Division of Stem Cell Transplantation, St Jude Children's Research Hospital, Mail stop 321, 332 N. Lauderdale St., Memphis, TN 38105, USA; 2 Children's University Hospital, Tuebingen, Hoppe-Seyler-Strasse 1, 72076 Tuebingen, Germany; 3 Children's University Hospital, Frankfurt, Theodor Stern Kai 7, 60580 Frankfurt, Germany

**Keywords:** neuroblastoma, high-dose chemotherapy, CD34 selection, lymphodepletion, T-cell homeostatic proliferation

## Abstract

We analysed the effect of graft-contaminating tumour cells on the long-term survival of 24 patients with high-risk neuroblastoma and found that patients whose grafts contained detectable neuroblastoma cells had a significantly higher probability of survival than did patients with no detectable tumour cells. Estimated contamination of the graft by more than 2000 tumour cells was associated with a significantly higher probability of survival than contamination with fewer tumour cells. We hypothesise that the presence of a critical number of graft-contaminating neuroblastoma cells can elicit a protective antitumour immune response after autologous transplantation.

Induction of a tumour-specific immune response is a major goal in the treatment of patients with solid tumours (Rosenberg, 2001), and promising clinical results have been obtained in the case of some malignancies (Jager *et al*, 2002). Most vaccination strategies have been clinically evaluated under conditions of steady-state haematopoiesis, but not during lymphoid reconstitution. However, the growth inhibition of sarcoma cells in mice during lymphopenia induced by sublethal total body irradiation is known for a long time (Hellstrom *et al*, 1978). Recently, a specific and long-lasting antitumour immune response was shown to be induced in RAG1 knockout mice by vaccination during early lymphoid reconstitution (Hu *et al*, 2002). In another study, greater activity against tumours was observed when mice were vaccinated immediately after myeloablative therapy and autologous bone marrow rescue (Borrello *et al*, 2000); similar approaches have been discussed for humans (Kwak, 1998).

A clinical scenario that most closely mimics those described in mice is autologous transplantation with a tumour-contaminated stem cell graft during chemotherapy-induced neutropenia and lymphopenia. Such a scenario is often encountered in children with high-risk neuroblastoma, up to 82% of whom have tumour contamination of their peripheral stem cell grafts (Lode *et al*, 1997; Leung *et al*, 1998; Burchill *et al*, 2001).

The observations described above suggested to us that the reinfusion of graft-contaminating tumour cells during chemotherapy-induced lymphopenia, and the subsequent reconstitution of lymphopoiesis, might exert a protective effect against relapse. We therefore retrospectively analysed the long-term survival and clinical course of 24 children with high-risk neuroblastoma who underwent autologous transplantation with stem cells purged by positive selection of CD34^+^ cells.

## PATIENTS AND METHODS

### Stem cell mobilisation and myeloablative therapy

A total of 24 consecutively treated children with high-risk neuroblastoma (23 with stage 4 disease and one with stage 3 disease with N-*myc* amplification) were included into the retrospective analysis. In all patients, peripheral blood stem cells (PBSC) were mobilised and collected as described (Klingebiel *et al*, 1995). Myeloablative therapy consisted of melphalan, etoposide, and carboplatin; some patients also had high-dose mIBG therapy, given as described (Klingebiel *et al*, 1998). Of the 24 patients, 21 received post-transplant immunotherapy with an antibody (ch14.18) to the tumour-associated disialoganglioside GD2 (Handgretinger *et al*, 2002). All transplants were given between January 1996 and February 1998. Median follow-up was 5 years.

### Stem cell purging and detection of residual neuroblastoma cells

The PBSCs of all patients were purged by positive selection of CD34^+^ stem cells by high-gradient magnetic-activated cell sorting (Handgretinger *et al*, 1998).The median purity of the CD34^+^ stem cells after positive selection was 97.6%. All 24 CD34^+^ stem cell grafts were examined for the presence of contaminating neuroblastoma cells by immunofluorescence with the chimeric anti-GD2 antibody delta ch14.18 (Handgretinger *et al*, 2002). This technique can detect contamination as low as one neuroblastoma cell in 10^5^ normal cells.

### Statistical analysis

Distribution of the probability of relapse-free survival was estimated by the method of Kaplan and Meier (1958). Differences in the estimated probability of survival were tested by using log-rank statistics. The number of tumour cells in the grafts of patients who did and did not experience relapse was compared by using the Wilcoxon rank-sum test. All tests were two-sided.

## RESULTS

**Figure 1:**
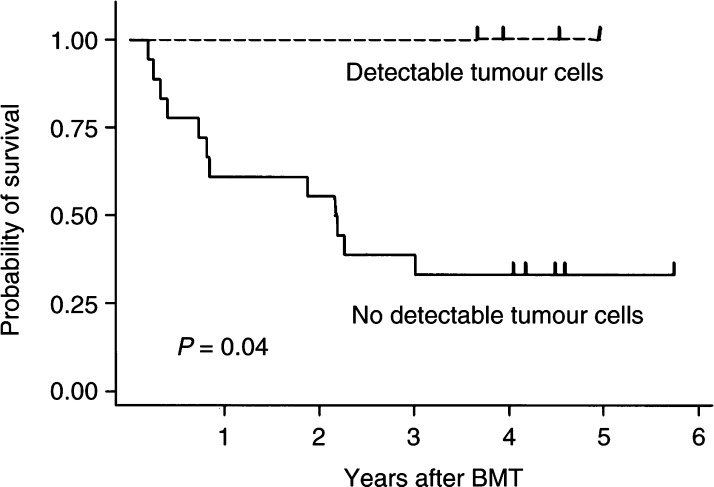
Kaplan–Meier analysis of the probability of relapse-free survival after autologous haematopoietic stem cell transplantation with and without detectable neuroblastoma cells in the graft.

The median number of transplanted purified CD34^+^ stem cells was 5.1 × 10^6^ kg^−1^ body weight (range 0.9–39.8 × 10^6^ kg^−1^) and the median to reach 0.5 × 10^9^ l^−1^ neutrophiles after myeloablative therapy was 12 days (range 8-24 days). Lymphoid reconstitution was analysed in eight patients and the median to reach >0.2 × 10^9^ and >1 × 10^9^ l^−1^ CD3^+^ T lymphocytes was 21 days and 6 months, respectively. Before purging, 17 of 22 grafts tested contained various numbers of tumour cells (0.82 × 10^6^–5.2 × 10^6^ tumour cells per graft). Tumour contamination was evaluated in all 24 grafts after purging. Four of the 24 grafts contained measurable neuroblastoma cells (a total of 2300–74 000 neuroblastoma cells per graft) and all four patients are long-term survivors after autografting. In contrast, the probability of 5-year survival among the other 20 patients was 33±11% (*P*=0.04, log-rank test) (Figure 1

). All deaths in this patient group were caused by relapse. These data suggest that graft-contaminating tumour cells induced a protective effect.

**Figure 2:**
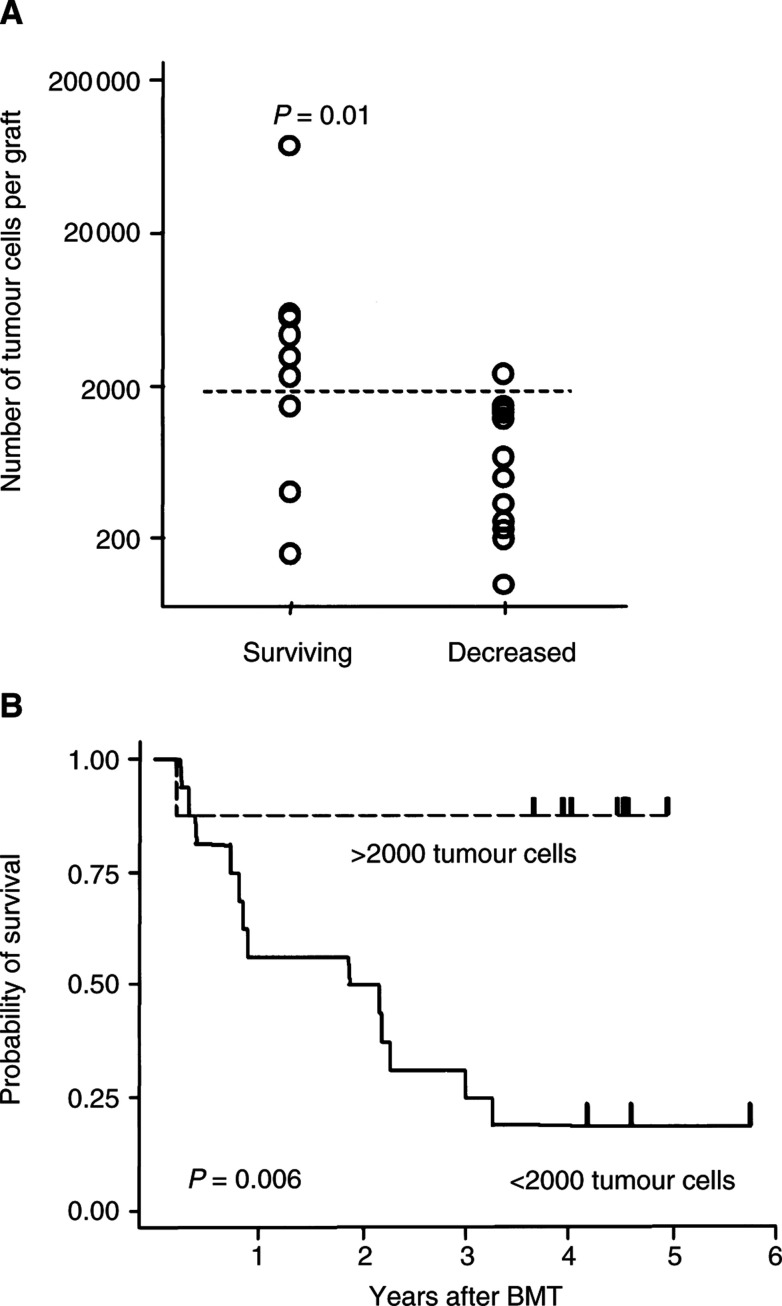
(**A**) Scatterplot analysis comparing the estimated absolute number of neuroblastoma cells in the autografts of patients who did and did not survive (*P*=0.01). (**B**) Comparison of relapse-free survival of patients whose autografts were estimated to contain more than 2000 cells or fewer than 2000 cells (*P*=0.006).

In order to estimate whether there would be a threshold number of protective graft-contaminating tumour cells, we calculated on the basis of the lower detection limit of our assay (one tumour cell per 10^5^ transplanted mononuclear cells) the possible absolute number of neuroblastoma cells in the 20 grafts that had no measurable tumour cells. We assumed maximal contamination based on our observation of tumour-cell contamination in 17 of the 22 evaluable unpurged grafts and the fact that more sensitive methods of tumour detection, such as polymerase chain reaction, often reveal the presence of tumour cells after purging (Lode *et al*, 1997; Leung *et al*, 1998). This estimation showed that survivors (*n*=10) had a significantly larger number of tumour cells in their grafts than did patients who died of relapse (*n*=14) (*P*=0.01, Wilcoxon rank-sum test, Figure 2A

). A scatterplot analysis also revealed a survival discriminant value of approximately 2000 tumour cells (Figure 2A). As shown in Figure 2B, the probability of 5-year survival was 88±12% for patients estimated to be infused with more than 2000 tumour cells (*n*=8) but only 19±10% for those estimated to be infused with fewer than 2000 tumour cells (*n*=16) (*P*=0.006, log-rank rest). The number of transplanted CD34^+^ stem cells or the remission status at the time of autologous transplantation had no influence on the outcome (data not shown).

## DISCUSSION

Our observations suggest that high-dose chemotherapy followed by a neuroblastoma-contaminated autograft induces an antineuroblastoma effect. The post-transplant lymphopenia induced by myeloablative chemotherapy and the subsequent homeostasis-driven proliferation of T cells may favour the induction of a tumour-specific immune response, as recently observed in mice (Dummer *et al*, 2002) and as discussed by Maine and Mule (2002). In addition, mice vaccinated with modified autologous tumour cells after transplantation had a higher probability of disease-free survival than did mice that were vaccinated in the absence of transplantation (Borrello *et al*, 2000). Moreover, tumour lysate-pulsed dendritic cells were able to elicit an effective antitumour immune response during early lymphoid recovery in a murine weakly immunogenic tumour model (Asavaroengchai *et al*, 2002). Altogether these findings suggest that exposure to tumour vaccines might be effective in inducing antitumour immunity during the post-transplant period of immune recovery. More recently, a high response rate has been reported in patients with metastatic melanoma after a nonmyeloablative lymphodepletion followed by adoptive transfer of autologous highly selected tumour-reactive T cells directed against overexpressed self-derived differentiation antigens (Dudley *et al*, 2002). The lymphodepletion in this setting may have led to disruption of homeostatic T-cell regulation or to abrogation of tolerogenic mechanisms. The reconstitution of CD3^+^ T lymphocytes occurred mainly during the first 6 months after autografting and is similar to published data on immune reconstitution after transplantation of highly purified autologous CD34^+^ stem cells (Heitger *et al*, 1999).

The use of purified CD34^+^ stem cells may offer an opportunity to reshape the repertoire of the reconstituting ‘naïve’ T lymphocytes to include tumour-associated self-antigens, thus overcoming any tumour-induced suppression of antitumour immunity (Sondak *et al*, 1991).

What could render autologous tumour cells immunogenic in the post-transplant period? Tumour-associated antigens (TAA), which are mostly tissue-specific or differentiation antigens encoded by normal genes (Boon and Old, 1997), are targets for a potential immune response (Nanda and Sercarz, 1995; Pardoll, 1999). TAAs, including NY-ESO-1, MAGE-1, and MAGE-3, have been described in neuroblastoma (Soling *et al*, 1999). In addition, it is conceivable that the graft-contaminating neuroblastoma cells are altered through the freezing and thawing process of the graft, which might render them immunogenic after infusion into the patient. It has been shown that cell freezing can induce stress proteins such as heat-shock proteins (Liu *et al*, 2000) and that heat-shock proteins can be bound and processed by dendritic cells (Schild and Rammensee, 2000). It has also been demonstrated that stressed apoptotic tumour cells can stimulate dendritic cells and induce specific cytotoxic T cells (Feng *et al*, 2002). The post-transplant treatment with the chimeric antibody ch14.18 may also have contributed, since antibody-induced apoptosis of neuroblastoma cells can also promote phagocytosis by dendritic cells and crosspriming of T cells, as described for an chimeric anti-CD20 antibody (Selenko *et al*, 2001).

Our observations suggest that graft-contaminating neuroblastoma cells induced a tumour-protective immune response after transplantation. If so, the initiation of such a response may have required a threshold quantity of tumour antigen. This would explain the association between long-term survival and the presence of more than 2000 estimated tumour cells in the graft. The reduction of the number of contaminating tumour cells by CD34^+^ cell selection might also play a role in survival by preventing the infusion of higher numbers of tumour cells that might overwhelm the immune system and result in relapse (Brenner *et al*, 1993). Unfortunately, this retrospective analysis did not allow us to investigate the expression of potential TAAs by patients' tumour cells. Although parts of our clinical observation were based on an assumption, our findings, taken with those of other investigators, provide a rationale for future trials in which autologous grafts of patients with high-risk neuroblastoma or other tumours are spiked with defined numbers of irradiated tumour cells or are vaccinated immediately after stem cell transplantation while still lymphopenic. A thorough analysis of the expression of potential TAAs by the individual patient's tumour cells and an analysis of the post-transplant immune response should reveal whether this approach results in an effective and long-lasting tumour protection.
